# Genomic, Proteomic, and Phenotypic Biomarkers of COVID-19 Severity: Protocol for a Retrospective Observational Study

**DOI:** 10.2196/50733

**Published:** 2024-02-14

**Authors:** Andrew English, Darren McDaid, Seodhna M Lynch, Joseph McLaughlin, Eamonn Cooper, Benjamin Wingfield, Martin Kelly, Manav Bhavsar, Victoria McGilligan, Rachelle E Irwin, Magda Bucholc, Shu-Dong Zhang, Priyank Shukla, Taranjit Singh Rai, Anthony J Bjourson, Elaine Murray, David S Gibson, Colum Walsh

**Affiliations:** 1 Personalised Medicine Centre, School of Medicine, Ulster University Derry/Londonderry United Kingdom; 2 National Horizons Centre Teesside University Middlesbrough United Kingdom; 3 Western Health Social Care Trust Londonderry United Kingdom; 4 Department of Biomedical and Clinical Sciences Linköping University Uppsala Sweden

**Keywords:** COVID-19, clinical research, multiomics, comorbidity, severity, electronic health record

## Abstract

**Background:**

Health organizations and countries around the world have found it difficult to control the spread of COVID-19. To minimize the future impact on the UK National Health Service and improve patient care, there is a pressing need to identify individuals who are at a higher risk of being hospitalized because of severe COVID-19. Early targeted work was successful in identifying angiotensin-converting enzyme-2 receptors and type II transmembrane serine protease dependency as drivers of severe infection. Although a targeted approach highlights key pathways, a multiomics approach will provide a clearer and more comprehensive picture of severe COVID-19 etiology and progression.

**Objective:**

The COVID-19 Response Study aims to carry out an integrated multiomics analysis to identify biomarkers in blood and saliva that could contribute to host susceptibility to SARS-CoV-2 and the development of severe COVID-19.

**Methods:**

The COVID-19 Response Study aims to recruit 1000 people who recovered from SARS-CoV-2 infection in both community and hospital settings on the island of Ireland. This protocol describes the retrospective observational study component carried out in Northern Ireland (NI; Cohort A); the Republic of Ireland cohort will be described separately. For all NI participants (n=519), SARS-CoV-2 infection has been confirmed by reverse transcription-quantitative polymerase chain reaction. A prospective Cohort B of 40 patients is also being followed up at 1, 3, 6, and 12 months postinfection to assess longitudinal symptom frequency and immune response. Data will be sourced from whole blood, saliva samples, and clinical data from the electronic care records, the general health questionnaire, and a 12-item general health questionnaire mental health survey. Saliva and blood samples were processed to extract DNA and RNA before whole-genome sequencing, RNA sequencing, DNA methylation analysis, microbiome analysis, 16S ribosomal RNA gene sequencing, and proteomic analysis were performed on the plasma. Multiomics data will be combined with clinical data to produce sensitive and specific prognostic models for severity risk.

**Results:**

An initial demographic and clinical profile of the NI Cohort A has been completed. A total of 249 hospitalized patients and 270 nonhospitalized patients were recruited, of whom 184 (64.3%) were female, and the mean age was 45.4 (SD 13) years. High levels of comorbidity were evident in the hospitalized cohort, with cardiovascular disease and metabolic and respiratory disorders being the most significant (*P*<.001), grouped according to the International Classification of Diseases 10 codes.

**Conclusions:**

This study will provide a comprehensive opportunity to study the mechanisms of COVID-19 severity in recontactable participants.

**International Registered Report Identifier (IRRID):**

DERR1-10.2196/50733

## Introduction

### Background

COVID-19 has a wide spectrum of clinical severity, with approximately 60% of cases thought to be asymptomatic or mildly symptomatic and approximately 5% being critically ill [1]. A severe infection is characterized by respiratory and multiorgan failure [2]. There are several known demographic risk factors, such as age, male sex, diabetes mellitus, and obesity [3], and recently, high-risk genes and genetic variation have gained extensive attention [4-8]. Identifying further biomarkers that reflect the pathophysiology of the disease and aid clinical staff in recognizing COVID-19 severity is critical [9]. This would also help in the development of clinical management systems that can improve patient outcomes [10]. Early work focused on easily accessible laboratory indices, such as elevated C-reactive protein and D-dimer, among others, which have been helpful in the early management of high-risk patients [9,11]. These biomarkers are commonly recorded in electronic care records (ECRs), a technological development that allows the exchange of health information electronically, facilitating effective diagnosis, reducing medical errors, and providing safer care and research [12]. However, the limitations of routine laboratory biomarkers are well documented [13].

Early work also implicated angiotensin-converting enzyme 2 (ACE2) receptors and type II transmembrane serine protease in viral entry [14,15]. A recent genome-wide association study of 2000 critically ill patients [5] identified dipeptidyl peptidase 9, tyrosine kinase 2, and the antiviral restriction enzyme activators OAS1, OAS2, OAS3. To date, singleomic approaches have been used to identify genomic markers of COVID-19 severity [5,16-18]. Here, we seek to use multiomics analysis using 2 tissue types (blood and saliva) in combination with comprehensive ECRs and self-reported data to build one of the most extensive pictures yet.

### Study Aims and Overview

The COVID-19 Response Study (COVRES; NCT05548829) aims to carry out an integrated multiomics analysis of factors contributing to host susceptibility to SARS-CoV-2 among a patient cohort of 1000 people from the geographically isolated island of Ireland. Because of differences in site, governance, and timelines, the protocol in the subsequent section describes the study to be carried out in Northern Ireland (NI-COVRES) by Ulster University and the Western Health and Social Care Trust (WHSCT) only. The Republic of Ireland component (Trinity College Dublin and St James Hospital Dublin) will be described separately.

**Figure 1:**
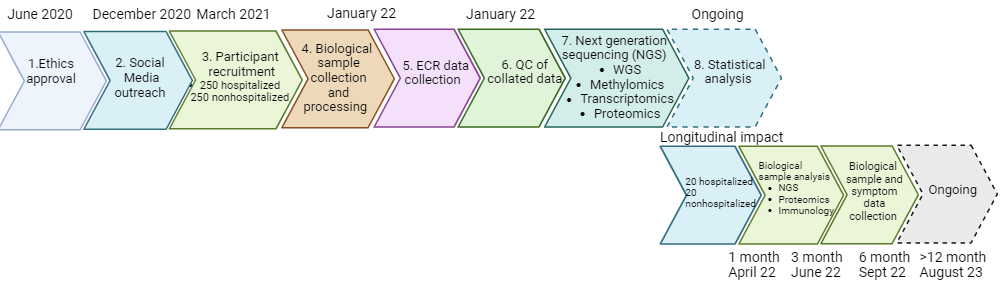
Stages of the COVID-19 Response Study implementation. ECR: electronic care record; NGS: next generation sequencing; QC: quality control; WGS: whole genome sequencing.

Figure 1 shows an overview of the main stages and timeline with data for each participant (n=519) on the following: (1) disease status, (2) genome analysis, (3) transcriptome analysis, (4) proteome analysis, (5) methylome analysis, (6) microbiome analysis, (7) immune response, (8) patient history, (9) mental health, and (10) ECR and prospectively on 40 participants at 1, 3, 6, and 12 months postpositive polymerase chain reaction (PCR) analysis to assess persistent inflammatory and immune responses.

## Methods

### Status and Timeline of the Study

**Figure 2:**
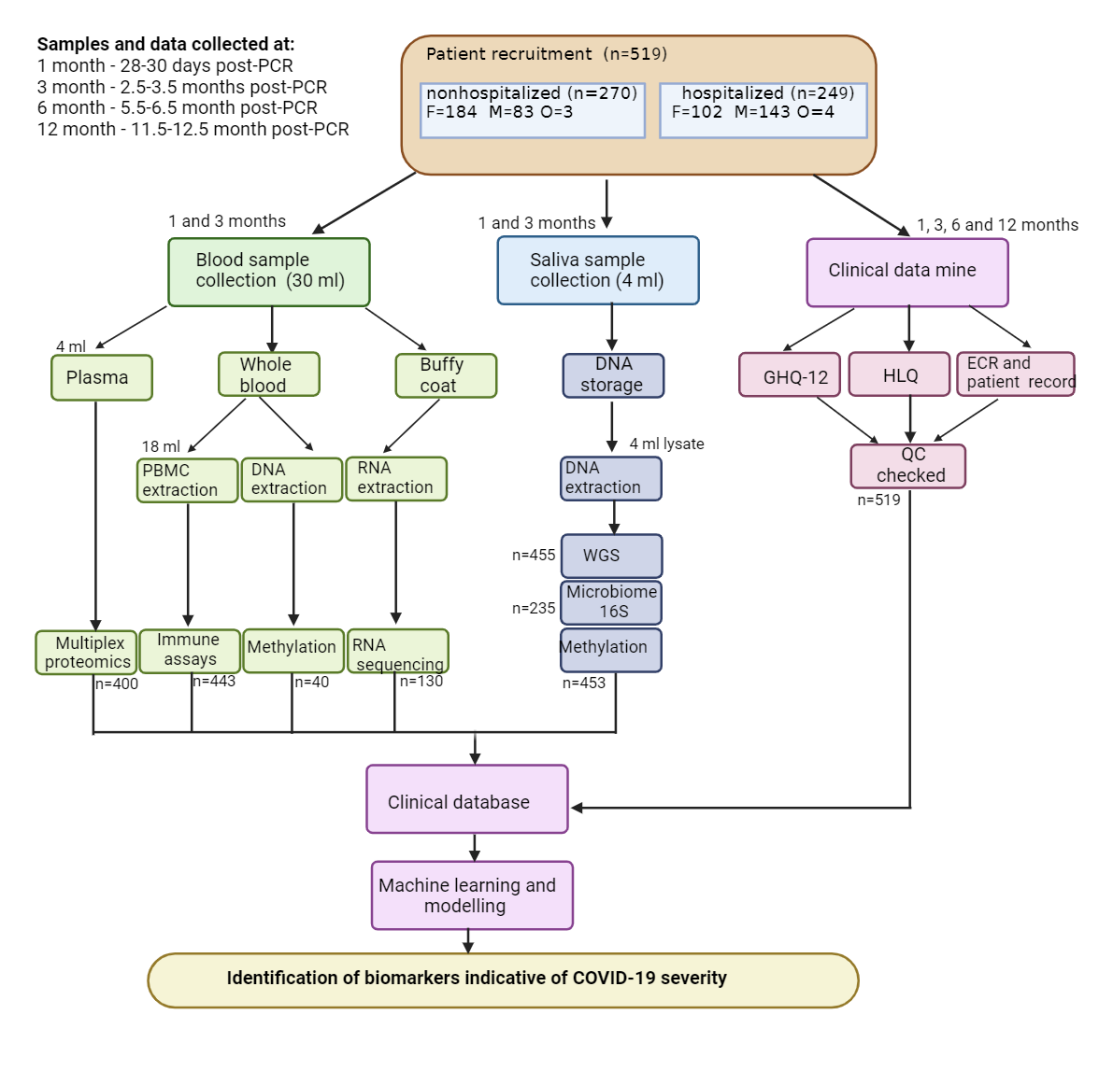
Overview of the COVID-19 Response Study sample processing work flow. ECR: electronic care record; FBC: full blood count; GHQ-12: 12-item General Health Questionnaire; HLQ: Health Life Style Questionnaire; MSD: Meso Scale Discovery; PBMC: peripheral blood mononuclear cell; PCR: polymerase chain reaction; TCR: T-cell receptor; QC: quality control; WGS: whole genome sequencing.

The main retrospective Cohort A recruitment commenced in December 2020 and was completed in March 2021 (trial registration; NCT05548829), except for the prospective Cohort B (ongoing). Integration of ECR record data was completed in January 2022 at the time of writing, and omics samples are being processed (Figure 2).

### Ethical Considerations

Standard operating procedures and participant response questionnaires included standard operating procedures for saliva sample kit preparation, blood collection and processing, downstream sample processing, website management, data protection and participant contact. The COVRES was subsequently approved by the Health and Care Research Wales Ethics Service on July 14, 2020 (Research Ethics Committee ref 20/WA/0179). All the participants provided informed consent to participate.

### Social Media Outreach

Social media content (on Twitter [subsequently rebranded X; Twitter, Inc] and Facebook [Meta Platforms, Inc]) and web page visuals were designed, with input from recovered patients, by the project principal investigators, including a range of infographics and short explanatory texts. Information was circulated to local and national news outlets (television, radio, and newspapers) across NI for recruitment purposes. Interested patients contacted the research team and were sent a patient information sheet. Appointments were then organized at least 24 hours later to obtain informed consent and samples.

### Participant Recruitment With Inclusion and Exclusion Criteria

Inclusion criteria: patients had to be aged >18 years but could have any BMI or ethnic origin. Exclusion criteria: patients were excluded if they were aged <18 years and had any intellectual disabilities. Hospitalization status was determined if a patient attended or was admitted to the hospital within 14 days of a positive PCR result. Patients were also classified based on the World Health Organization (WHO) scale [19], which reflects severity over the duration of the patient’s infection, regardless of hospitalization status. For example, a patient may have an overall WHO score of 5 and be classified as nonhospitalized as they attended the hospital >14 days from their positive PCR result. After receiving a participant information sheet, patients interested in participating provided written informed consent and were enrolled in the study. A self-report questionnaire established demographic information, lifestyle choices, family history of clinical disorders, and COVID-19 severity and symptoms. This was followed by a 12-item general health questionnaire to help ascertain the patient’s mental health after the COVID-19 infection (Figure 2). This data was securely digitalized onto a bespoke database, CovresNIdb, generated on the REDCap (Research Electronic Data Capture; Vanderbilt University) platform [19], to comply with the terms of the ethical considerations, the Human Tissue Act 2004, and general data protection regulations. This process is being repeated for Cohort B (prospective), consisting of 40 participants with stricter timelines followed (1, 3, 6, and 12 mo).

### Biological Sample Processing

The WHSCT recruitment team coordinated sample collection appointments at hospital wards, Clinical Translational Research and Innovation Centre clinic rooms, or home visits. Participants and related study code numbers were predetermined depending on hospitalization and logged into encrypted clinical data sheets on a secure server to ensure full data traceability. All whole blood and saliva processing carried out includes recruitment numbers, sample collection types, sample processing, and downstream analysis; n numbers refer to patient numbers for specific omics analyses.

Isolation was carried out in a Category III containment hood with full personal protective equipment. Samples were not deactivated upon receipt or before processing. The participants provided 3×10 mL of whole blood and 2× saliva samples of approximately 2 mL each (Figure 2). Blood was extracted using 21G Vacuette safety needles (Greiner Bio-One Ltd, Gloucestershire) into 3×10 mL ethylenediaminetetraacetic acid–coated Vacuette tubes and centrifuged at 4000 rpm (4 °C) for 15 minutes. The buffy coat was extracted, washed, and stored for RNA sequencing (Figure 2). All samples were frozen at −80 °C; the time to freeze was <2 hours, and none showed signs of hemolysis. Saliva was collected using 1×DNA Genotek (DNA Genotek, Ottawa), Oragene DNA (OG-500), and 1×RNA (CP-190) collection tubes per participant. Samples were considered deactivated once lysed. Peripheral blood mononuclear cells were isolated using the Ficoll gradient separation methods as per [20].

### Immune Assays

Whole blood was analyzed at 1 and 3 months post–positive PCR tests. Using the FACSAria III high-speed cell sorter (Becton Dickinson, Oxford, United Kingdom, software version 9) with an 85-µm nozzle fitted, whole blood and peripheral blood mononuclear cell samples were stained for T, B, and natural killer cell populations using CD45 PerCP-Cy5.5, CD3 FITC, CD8 APC-Cy7, CD4 PE-Cy7, CD19 APC, and CD16+CD56 PE (BD Biosciences) before erythrocyte lysis by PharmLyse (BD Biosciences) according to the manufacturer’s instructions. T-cell subpopulations were measured using 2 defined panels: panel 1: CD3 FITC, CD4 PE-Cy7, CD8 BV605, CD30 APC, CD45RA V450, CD45RO BV786, and CD183 BB700; panel 2: CD3 FITC, CD4 PE-Cy7, CD8 BV605, CD69 APC, CD45 V450, CD127 BV786, CD152 BB700, CD25 R718, and FoxP3 PE. Cell surface staining was performed before fixing, permeabilizing, and FoxP3 labeling using the Transcription Factor Buffer Set (BD Pharmingen).

### DNA Isolation

Saliva samples (whole-genome sequencing [WGS], methylome, and microbiome) were incubated for 2 hours at 56 °C, followed by DNA isolation using PrepIT L2P (DNA Genotek). DNA from whole blood (methylome) was isolated using the DNA Blood 200,360 prefilling H96 Kit (CMG-717, PerkinElmer), and 200 µL of whole blood on the Chemagic 360 system (PerkinElmer) was used. Microbial DNA was extracted from saliva aliquots using a modified protocol from Teng et al [21] using the DNeasy Blood and Tissue Kit (Qiagen). All extracted DNA was evaluated using the Qubit 3.0 fluorometer (Thermo Scientific) and the Nano Drop 1000 spectrophotometer (Thermo Scientific) and sequenced using the Invitrogen Quant-iT PicoGreen dsDNA Assay Kit (P7589) on the Hamilton Microlab Star before storage at −80 °C.

### RNA Isolation

RNA was isolated from saliva using the Oragene RNA purification protocol and Qiagen RNeasy Micro Kit (Qiagen), and RNA was extracted from whole blood using the Chemagic 360 system (PerkinElmer) with the Chemagic RNA tissue 360 H96 Kit (CMG-1212). Purity and quantity were assessed, as described in the preceding section for DNA, but with the Invitrogen Quant-iT RiboGreen Assay Kit (R11490). Integrity (RIN) was determined using the Agilent 4200 TapeStation and the RNA ScreenTape (5067-5366), before storage at −80 °C.

### Clinical Data

#### Self-Reported Data on Physical and Mental Well-Being

All participants completed 2 surveys as part of the trial. The 12-item general health questionnaire is a self-administered screening tool designed to detect current mental state disturbances in primary care settings; a score of ≥2 indicates a disorder. The health and lifestyle questionnaire is a survey tool designed by Ulster University to capture key health-related data not present in the ECR. The fields included COVID-19 risk factors, medications, comorbidities, hospitalization information, symptoms at admission, laboratory tests, family history, drinking status, and occupation. The same protocol is being followed for all prospective appointments (ongoing).

#### Clinical Database Development

The participants’ consent forms, as well as data from the self-reported questionnaires, but with all personally identifiable information removed by the project’s data controller as per general data protection regulations guidelines, were also recorded in the CovresNIdb database. The data were subjected to quality control (QC) by 2 independent researchers against the original sources. The same protocol is being followed for all prospective appointments (ongoing).

#### Electronic Care Records

In addition to the self-reported data, consent was also provided by each patient to enrich the database by accessing their NI Electronic Care Record information held by the National Health Service. PCR positive dates, severity (hospitalized because of COVID-19 infection or recovered from COVID-19 infection at home), laboratory results (full blood count, blood pressure, lipids, C-reactive protein, glomerular filtration rate, and troponin), treatment administered, drugs prescribed within the last 6 months, and comorbidity and multimorbidity were recorded for each patient.

### Omics Analyses

#### Genome Analysis

Whole-genome library preparation was performed using the Illumina TruSeq PCR Free Library Prep protocol (20015963) with an input amount of 1 µg on a Hamilton next-generation sequencing Star robotic workstation, and quality was assessed using the Roche KAPA Library Quantification Kit (7960298001) before pooling and sequencing (150bp PE) on an Illumina NovaSeq 6000 instrument using NovaSeq 6000 S4 Reagent Kit v1.5 (20028312), with a mean coverage of 30× as described previously [22]. Sequences are being uploaded to the European Genome-phenome Archive (EGA).

#### Methylome Analysis

Methylation analysis was performed on DNA samples from saliva (n=450) and whole blood (n=40) using the Illumina Infinium Methylation EPIC, largely as described previously [22]. Data were adjusted for known epigenetic covariates, and surrogate variable analysis was performed via the *sva* inference module [23]. Our in-house-developed tool, CandiMeth [24], will be used to streamline the methylation analysis of gene lists of interest.

#### Transcriptome Analysis

RNA sequencing library preparation was performed using the Illumina TruSeq Stranded Total RNA Library Prep Globin Kit (20020612) with an input amount of 100 to 1000 ng. Library preparation was automated and processed using a Hamilton next-generation sequencing Star, and quality was assessed using the Roche KAPA Library Quantification Kit (7960298001) and GX Caliper HS Assay (CLS 760,672, 760,517), run on Roche Lightcycler 480 II and PerkinElmer LabChip GX Touch analyzers, respectively. Libraries were pooled and sequenced (75bp PE) on an Illumina NovaSeq 6000 instrument using NovaSeq 6000 S2 Reagent Kit v1.5 (20028314), targeting 50M paired reads. Raw data binary base call format was demultiplexed and converted to the FASTQ format using BCL2FastQ (Illumina). Adapters were trimmed using Skewer [25], and QC was assessed using FASTQC. Spliced Transcripts Alignment to a Reference [26] was used to align reads to the reference genome (GRCh38/hg38) as well as to the transcriptome (version 25; GENCODE). The quality of the RNA alignment was assessed using Picard QC. Gene and isoform quantification will be performed using RNA-Seq by Expectation-Maximization [27], with prospective patient (1 and 3 mo) T-cell receptor sequencing completed following flow cytometry.

#### Microbiome Analysis

16S ribosomal RNA gene amplicons for sequencing by the Illumina MiSeq system (Illumina) were prepared using the V3 and V4 regions as described by Klindworth et al (2013), with sequencing performed in-house.

#### Proteome Analysis

Protein analysis of 400 plasma samples (baseline; 186 nonhospitalized and 214 hospitalized), 40 prospective (20 nonhospitalized and 20 hospitalized; 1 and 3 months postinfection), was outsourced to OLINK proteomics (OLINK, Uppsala, Sweden) using the Explore 384 Inflammation panel (Protein Proximity Extension Assay). Ethylenediaminetetraacetic acid plasma samples were thawed at room temperature (20 °C), and 45 µL of each plasma sample was (at random) pipetted into a LightCycle 480Multiwell Plate 96-well white PCR plates (product no. 04729692001; Roche Molecular Systems Inc) with 8 × wells left empty on each plate for internal controls to be added at OLINK. The samples were inactivated as per OLINK’s protocol and shipped on dry ice (CO2, −78 °C). Only samples above 0.2 Normalized Protein eXpression and samples that deviated less than 0.3 Normalized Protein eXpression passed QC.

The Meso Scale Discovery plasma multispot assay system comprising V-PLEX COVID-19 serology panel 11, *total IgG*, and *ACE2 neutralization* assays was used to determine viral variant prevalence. Samples were prepared at 1:10 (ACE2) and 1:5000 (neutralization) for specific assays and then treated as described in [28].

The Roche COBAS Elecsys SARS-CoV-2 spike (S) protein receptor binding domain assay was used to determine SARS-CoV-2 antibody presence as per manufacturer’s instructions.

### Statistics

#### Univariate and Multivariate Analysis

Only patients from Cohort A (n=507) who had their BMI recorded in the database were selected for the odds ratio analysis. We considered the following risk factors: sex, age, BMI, and disease subgroups. First, univariate analyses (Fisher exact test) were performed to identify risk factors associated with COVID-19 severity.

*P* values for univariate analyses were generated using the Fisher exact test to compare the frequencies of each potential risk factor between nonhospitalized and hospitalized participants. Variables with a *P* value <.001, that is, sex, age <50 years and >50 years, and cardiovascular, respiratory, endocrine, and musculoskeletal comorbidities, were considered clinically relevant and entered into the multivariable logistic regression model. This and further analysis are being undertaken on Base-R software (R Project for Statistical Computing; version 4.2.2) using the Visdat library.

#### Demographics Table

The demographic table of the COVRES data (n=519) was generated using IBM SPSS Statistics for Windows (version 27; IBM Corp) [29]. Statistical analysis for the contingency table was undertaken using a Fisher exact 2-sided test to obtain the required *P* values, and CI rates were set at 95%.

#### Bioinformatic Analyses

Bioinformatic analyses will focus on using computational approaches to identify genomic, transcriptomic, proteomic, and clinical correlates of severity. Planned analyses primarily include the identification of clinical features, gene variants (host) or Expression quantitative trait loci, transcriptomic signatures, and cytokine profiles associated with disease severity, as well as the differential methylation among the host genomes of the severity groups.

Variant calling will use mathematical models from the Best Practices Genome Analysis Toolkit. Data are being stored according to genomic position in the Genuity Science Genomically Ordered Relational Database to facilitate rapid access by the Clinical Sequence Analyzer user interface and Sequence Miner visualization software. Initial data processing for methylome analysis will be carried out in *GenomeStudio* (version 3.2; Illumina) before the import of idat files into the RnBeads package [30] using RStudio (version 2022.02.0+443) on the R platform (version 4.1.2). QC will be performed using the *greedycut* algorithm, which involves the removal of probes with missing values and poor quality. For RNA-seq, gene and isoform quantification will be performed using RNA-Seq by Expectation-Maximization [27] before further analysis is carried out. 16S ribosomal RNA analysis has been previously described (refer to the preceding section), and OLINK data will be processed in R as per standard pipelines. WGS and transcriptomics data are to be deposited in the EGA pending and shared in collaboration with the International COVID-19 Host Genetics Initiative.

## Results

### Retrospective Cohort A Demographics

The main demographic characteristics are summarized in Table 1.

**Table 1:** COVID-19 Response Study Cohort A demographic information.

Cohort A demographics			Nonhospitalized (n=270)		Hospitalized (n=249)		Total (n=519)		*P* value^a^
**Sex, n (%)**									
	Female	184 (64.3)		102 (35.7)		286 (55.1)		<.001	
	Male	83 (36.7)		143 (63.3)		226 (43.5)		<.001	
	Other	3 (42.9)		4 (57.1)		7 (1.3)		.72	
**Age (y) at diagnosis**									
	Age, mean (SD)	45.4 (13)		56.5 (12.7)		50.7 (14)		<.001^a^	
	<50, n (%)	169 (62.6)		67 (26.9)		236 (45.5)		<.001	
	>50, n (%)	101 (37.4)		182 (73.1)		283 (54.5)		<.001	
**Disease subgroup, n (%)**									
	Autoimmune^b^	12 (4.4)		26 (10.4)		38 (7.3)		.01	
	Metabolic^c^	33 (12.2)		94 (37.8)		127 (24.5)		<.001	
	Respiratory^d^	39 (14.4)		83 (33.3)		122 (23.5)		<.001	
	Cardiovascular^e^	32 (11.9)		100 (40.2)		132 (25.4)		<.001	
	Cancer^f^	7 (2.6)		21 (8.4)		28 (5.4)		.003	
	Gastrointestinal^g^	13 (4.8)		21 (8.4)		34 (6.6)		.11	
	Musculoskeletal^h^	23 (8.5)		58 (23.3)		81 (15.6)		<.001	

^a^*P* value calculated using 2-sided Fisher exact test between nonhospitalized versus hospitalized patients. *P*<.05 was set as statistically significant (n=519); continuous variables used a 2-sided *t* test.

^b^Autoimmune or rheumatic diseases including rheumatoid arthritis, systemic lupus erythematosus, and multiple sclerosis.

^c^Metabolic and endocrine diseases including thyroid conditions, hypercholesterolemia, or other hyperlipidemia, gout, diabetes, and kidney disorders.

^d^Respiratory disorder and chronic lung diseases including chronic obstructive pulmonary disease, asthma (moderate to severe), interstitial lung disease, cystic fibrosis, sleep apnea, and pulmonary hypertension.

^e^Cardiovascular system disorders including angina, hypertension, stroke, peripheral vascular disease, balloon angioplasty or percutaneous coronary intervention, atrial fibrillation, venous thromboembolism, anemia, and chronic cardiac diseases other than hypertension.

^f^Cancer including leukemia, lymphoma, and malignant solid tumors, and to include current, past, and remission.

^g^Gastrointestinal disorders including gallbladder, liver disease, pancreatic disease, and Inflammatory bowel syndrome.

^h^Musculoskeletal diseases including osteoarthritis and ankylosing spondylitis, excluding subgroup 1 conditions.

As expected, there was a significant difference in the mean age between hospitalized and nonhospitalized patients, as well as between sexes (both *P*<.001). Age bias was also evident, with 62.6% (169/270) of patients aged <50 years in the nonhospitalized subgroup (*P*<.001) and 73.1% (182/249) of patients aged >50 years in the hospitalized subgroup (*P*<.001). As expected, comorbidity incidence was higher in the hospitalized subgroup than in the nonhospitalized subgroup, with autoimmune (12/270, 4.4% nonhospitalized and 26/249, 10.4% hospitalized; *P*<.001), metabolic (33/270, 12.2% nonhospitalized and 94/249, 37.8% hospitalized; *P*<.001), respiratory (39/270, 14.4% nonhospitalized and 83/249, 33.3% hospitalized; *P*<.001), cardiovascular (32/270, 11.9% nonhospitalized and 100/249, 40.2% hospitalized; *P*<.001), and musculoskeletal (23/270, 8.5% nonhospitalized and 58/249, 23.3% hospitalized; *P*<.001) disorders of note. There were no differences between the cohorts for gastrointestinal disorders (Table 1).

### Prospective Cohort B Demographics

Data collection is ongoing for 40 participants who are being followed up over 12 months: 20 (8/20, 40% female) hospitalized and 20 nonhospitalized (12/20, 60% female); sex distribution is not significantly different between subgroups (*P*=.21) but average age is (hospitalized: mean 52, SD 17.2 years; nonhospitalized: mean 45.2, SD 13.5 years; *P*<.001; Table 2).

In contrast to the initial recruitment, within this follow-up Cohort B, there was no age bias toward participants older than 50 years (*P*=.20). There was also no significant difference in vaccination status between hospitalization subgroups (*P*=.55), and only cardiovascular disease as a comorbidity was more prevalent in hospitalized patients (*P*=.02), although the numbers are small.

**Table 2:** COVID-19 Response Study Cohort B multivariate analysis of hospitalized versus nonhospitalized patients analyzing risk factors for COVID-19 severity.

Prospective Cohort B demographics		Nonhospitalized (n=20)	Hospitalized (n=20)	Total (n=40)	*P* value^a^
Female, n (%)		12 (60)	8 (40)	22 (50)	.21
**Age (y) at diagnosis**					
	Age, mean (SD)	45.2 (13.5)	52 (17.2)	48.6 (15.6)	<.001^b^
	>50, n (%)	7 (35)	11 (55)	18 (45)	.20
Vaccine status, n (%)		19 (95)	18 (90)	37 (93)	.55
**Comorbidity, n (%)**					
	Autoimmune^c^	3 (15)	6 (30)	9 (23)	.26
	Metabolic^d^	4 (20)	9 (45)	13 (33)	.91
	Respiratory^e^	2 (10)	7 (35)	9 (26)	.58
	Cardiovascular^f^	2 (10)	11 (55)	13 (33)	.02
	Cancer^g^	0 (0)	4 (20)	4 (10)	.35
	Gastrointestinal^h^	2 (10)	4 (20)	6 (15)	.38
	Musculoskeletal^i^	3 (15)	7 (35)	10 (25)	.14

^a^*P* value calculated using 2-sided Fisher exact test between nonhospitalized versus hospitalized patients. *P*<.05 set as statistically significant (n=40); continuous variables used a 2-sided *t* test.

^b^Inclusion criteria for analysis: participants (n=40) were required to have a BMI score recorded.

^c^Autoimmune or rheumatic diseases including rheumatoid arthritis, systemic lupus erythematosus, and multiple sclerosis.

^d^Metabolic and endocrine diseases including thyroid conditions, hypercholesterolemia, or other hyperlipidemia, gout, diabetes, and kidney disorders.

^e^Respiratory disorder and chronic lung diseases including chronic obstructive pulmonary disease, asthma (moderate to severe), interstitial lung disease, cystic fibrosis, sleep apnea, and pulmonary hypertension.

^f^Cardiovascular system disorders including angina, hypertension, stroke, peripheral vascular disease, balloon angioplasty or percutaneous coronary intervention, atrial fibrillation, venous thromboembolism, anemia, and chronic cardiac diseases other than hypertension.

^g^Cancer including leukemia, lymphoma, and malignant solid tumors, and to include current, past, and remission.

^h^Gastrointestinal disorders including gallbladder, liver disease, pancreatic disease, and Inflammatory bowel syndrome.

^i^Musculoskeletal diseases including osteoarthritis and ankylosing spondylitis, excluding subgroup 1 conditions.

## Discussion

### Principal Findings

The COVRES provides a novel opportunity to identify multiomics biomarkers from blood and saliva indicative of COVID-19 severity in NI and may provide unique insights into disease mechanisms and identify potential therapeutic targets. The maximum recruitment number (n=519) was reached, and various analyses are ongoing, including WGS and RNA sequencing, proteomic, microbiome, and methylation experiments. We have also collected detailed medical data using the NI Electronic Care Record that will be used to enrich the biomarker data [31]. All Cohort A participants were recruited over a 4-month period (December 1, 2020-March 31, 2021) during the pandemic peak, allowing homogeneous data collection from the same viral variant (B.1.1.7; Figure 1). Compared with other studies, the COVRES has a higher participant number and uses a significantly wider biomarker identification approach. This was achieved while significant pandemic restrictions were in place and was only possible because of our local health trust (WHSCT, National Health Service) collaboration, which facilitated patient access and enabled the recording of laboratory parameters that have not been possible in other studies [32]. A recent multiomics COVID-19 study used proteomics and metabolomics to screen 13 samples at 2 time points and found 10 significant proteins, 32 significant peptides, and 5 metabolites that were dysregulated in severe patients [33]. Recruitment for this study also occurred in early 2021, but the small sample size (n=13) brings into question the generalizability of the findings. Another multiomics study based in the United States sampled 128 individuals between April 6, 2020, and May 1, 2020, and conducted follow-up until June 2020. The authors quantified transcripts, proteins, metabolites, and lipids and made associations with clinical outcomes [34]. Links were made between platelet function, blood coagulation, endotheliopathy, and COVID-19 severity. Our study builds on these smaller studies and may offer increased statistical power and the potential to validate or compare the markers identified.

The COVRES was designed to recruit hospitalized (n=250) patients with COVID-19, classified as having a severe infection, and nonhospitalized (n=250) patients with COVID-19, classified as having a mild infection, within 3 months of sampling. It is worth noting that the recruitment of nonhospitalized patients with COVID-19 makes this cohort particularly valuable, as most trials have only involved patients who have been admitted to the hospital or those who have not [35,36], and few have investigated earlier stages of the disease process, such as preexposure or postexposure and outpatient treatment.

To maximize the impact and benefit to the scientific and health care communities, this study was designed to be cross-border, covering both NI and the Republic of Ireland. The global drive to identify clinical biomarkers of COVID-19 severity has led to many clinical studies and trials that have varied methodologies regarding different control groups, follow-up periods, omics of interest, and laboratory methodologies [37-39]. Studies have also been carried out in different geographic regions without any standardized operating procedures and have been powered according to different end points [40]. This variation makes reproducibility questionable, and it is difficult to apply the findings across geographic regions and variant periods. To align with as many studies as possible, COVRES participants have been classified according to the WHO [41], and to facilitate cross-border collaboration, we coordinated with Trinity College Dublin. We also plan to share our WGS data with the EGA for the advancement of science and improved public health outcomes.

The recruitment of nonhospitalized and hospitalized patients with COVID-19 in NI is the main strength of the COVRES and adds novelty to existing research regarding COVID-19 severity, with the majority recruiting patients based on a positive PCR test result regardless of hospitalization. Sex and age matching was considered, but an exact match was not achieved because of the complexities and limitations of COVID-19 presented regarding patient access [5]. The mean age of the hospitalized COVRES subgroup was 56.5 years (Table 1), which was slightly younger compared with a large UK-wide observational study [42] (20,908 hospitalized), which had a mean age of 62 years. There was no difference between sex (male 49%, female 51%), compared with our sample comprising 43.5% (226/519) men. Another smaller (n=429) UK study found the average age of hospitalized patients with COVID-19 to be 70 years and a male bias of 57%, which is close to our study. Corresponding with our study, they also found the average BMI to be 28 kg/m^2^ (overweight-obese) and highly comorbid (Table 2), with the most common comorbidities being type-2 diabetes, hypertension, and respiratory disorders [42,43]. A previous study [43] in England is a good comparison for COVRES NI, as the recruitment protocols and cohort demographics are similar. The similarities in the data are promising and may indicate that our findings could be useful to the wider United Kingdom.

### Limitations

COVRES participants were all sampled at a single time point, limiting our ability to assess genomic, proteomic, and immune biomarkers as the disease progresses. Future work will focus on obtaining follow-up samples to enable longitudinal analysis and assess the prognostic capability of markers of interest. Manual data input at some points increases the risk of human error [44], and although QC checks were carried out between 2 WHSCT staff members, there is an inherent risk of incorrect data.

It also needs to be considered that the COVRES cohort represents a COVID-19 population recruited in NI, and the demographics show a low representation of ethnic minority groups; therefore, data may not be able to be generalizable beyond White Irish and UK populations.

### Conclusions

The COVRES offers a novel opportunity to study the multiomics mechanisms of COVID-19 severity in recontactable participants. This research has the potential to impact COVID-19 clinical decision-making and therapeutic development. Our WHSCT and industry collaborators enabled rapid and effective recruitment, allowing us to reach our goal of 500 participants and begin the analysis pipelines immediately. We hope that this paper will not only demonstrate the effectiveness of the study methodology but also raise awareness of the availability of this cohort among researchers in the field and promote future collaboration.

## Abbreviations

ACE2: angiotensin-converting enzyme 2
COVRES: COVID-19 Response Study
ECR: electronic care record
EGA: European Genome-phenome Archive
NI: Northern Ireland
PCR: polymerase chain reaction
QC: quality control
REDCap: Research Electronic Data Capture
WGS: whole-genome sequencing
WHO: World Health Organization
WHSCT: Western Health and Social Care Trust

## Data Availability

Data and metadata will be stored according to the Ulster University policy on data management and sharing. Data will be available via Ulster University’s Research Data Repository and in accordance with their Research Data Management Policy. Any personal and identifiable information will be redacted, and data queries will be addressed on an individual basis by the research team. Genomics data are being deposited in the European Genome-phenome Archive and will be available upon request.
